# Supplementary material for: Experimental impacts of grazing on grassland biodiversity and function are explained by aridity
Source: Nat Commun. 2023 Aug 19;14:5040. doi: 10.1038/s41467-023-40809-6 (PMC10439935; doi:10.1038/s41467-023-40809-6)
Supplement: Supplementary file 1 — Supplementary Information [file 41467_2023_40809_MOESM1_ESM.pdf]

Experimental impacts of grazing on grassland biodiversity and function are explained by aridity

Minna Zhang[1], Manuel Delgado-Baquerizo[2, 3], Guangyin Li[1, 4], Forest Isbell[5], Yue Wang[1], Yann Hautier[6], Yao Wang[1], Yingli Xiao[1], Jinting Cai[1], Xiaobin Pan[1], Ling Wang[1*]

[1]*Institute of Grassland Science, Key Laboratory of Vegetation Ecology of the Ministry of Education, Jilin Songnen Grassland Ecosystem National Observation and Research Station, Northeast Normal University, Changchun 130024, China.*

[2]*Laboratorio de Biodiversidad y Funcionamiento Ecosistémico. Instituto de Recursos Naturales y Agrobiología de Sevilla (IRNAS), CSIC, Av. Reina Mercedes 10, E-41012, Sevilla, Spain.*

[3]*Unidad Asociada CSIC-UPO (BioFun). Universidad Pablo de Olavide, 41013 Sevilla, Spain.*

[4]*Key Laboratory of Wetland Ecology and Environment, Heilongjiang Xingkai Lake Wetland Ecosystem National Observation and Research Station, Northeast Institute of Geography and Agroecology, Chinese Academy of Sciences, Changchun 130102, China.*

[5]*Department of Ecology, Evolution and Behavior, University of Minnesota, 1479 Gortner Ave., Saint Paul, MN, USA.*

[6]*Ecology and Biodiversity Group, Department of Biology, Utrecht University, Utrecht, the Netherlands.*

Corresponding author: Ling Wang

Email: wangl890@nenu.edu.cn

Tel: +86–431–85099737; Fax: +86–431–85695065

Table S1. Characteristics of the geographic, climatic and plant variables at the study sites across the temperate grasslands of northern China.

Site	Latitude	Longitude	Aridity	MAP (mm)	MAT (°C)	Elevation (m)	Grassland type	Exclosure year	Dominant plant species
1	49.52	120.03	0.438	398	-2	808	Meadow steppe	14	*F. sibiricum*
2	49.35	120.13	0.482	384	-1.5	668	Meadow steppe	19	*L. chinensis, S. baicalensis*
3	49.33	120.05	0.497	374	-1.0	626	Meadow steppe	17	*L. chinensis*
4	44.59	123.51	0.509	435	5.4	180	Meadow steppe	20	*L. chinensis*
5	48.50	119.68	0.511	361	-0.9	753	Meadow steppe	19	*S. baicalensis*
6	43.55	116.69	0.578	346	1.1	1249	Typical steppe	23	*L. chinensis*
7	43.60	116.75	0.586	346	1.1	1208	Typical steppe	38	*S. grandis*
8	44.15	116.35	0.648	308	1.2	1097	Typical steppe	12	*S. grandis, L. chinensis*
9	41.25	111.23	0.626	306	2.6	1703	Desert steppe	10	*S. breviflora, S. krylovii*
10	41.79	111.90	0.746	232	3.3	1435	Desert steppe	19	*S. breviflora*

Table. S2. The theoretical information on all 11 functions and their importance.

Ecosystem functions	Importance
Plant community N	The key ecosystem processes that sustain human welfare, supply important nutrient sources for livestock herbivores (e.g., proteins and energy), and plays major roles in the global carbon cycle.
Plant community P	
Above-ground plant biomass	
Below-ground root biomass	A key ecosystem process that supports belowground functionality.
*S*oil available N	The fraction of the soil N pool that is more readily available for plant and microbial uptake.
Soil organic C	Soil carbon-fixation function, climate regulation.
Microbial biomass C	Reflecting the dynamics of soil quality and microbial activities.
Microbial biomass N	
Relative abundance of saprotrophs in soils	Decomposition.
Reduced relative abundance of fungal plant pathogens in soils	Pathogen control.
Relative abundance of mutualistic fungi in soils	Mycorrhizal colonization.

Table. S3. Summary of linear mixed effects models analyzing the interactive effects of grazing and grassland types on ecosystem multifunctionality, multidiversity, above-ground diversity, below-ground diversity, multirichness, above-ground species richness, and below-ground species richness (two-tailed statistical tests). Grazing, grassland types, and their interaction were taken as fixed factors. Plot nested within sites were taken as random factor. Source data are provided as a Source Data file.

	Variable	DF	F-value	p-value
Ecosystem multifunctionality	Grazing	47	10.574	**0.002**
	Grassland types	7	3.257	0.100
	Grazing: Grassland types	47	4.997	**0.011**
Multidiversity	Grazing	47	1.026	0.316
	Grassland types	7	7.737	**0.017**
	Grazing: Grassland types	47	5.052	**0.010**
Above-ground diversity	Grazing	47	16.760	**<0.001**
	Grassland types	7	0.927	0.439
	Grazing: Grassland types	47	5.758	**0.006**
Below-ground diversity	Grazing	47	5.335	**0.025**
	Grassland types	7	11.279	**0.007**
	Grazing: Grassland types	47	4.016	**0.025**
Multirichness	Grazing	47	9.538	**0.003**
	Grassland types	7	5.175	**0.042**
	Grazing: Grassland types	47	2.193	0.123
Above-ground species richness	Grazing	47	15.039	**<0.001**
	Grassland types	7	1.249	0.344
	Grazing: Grassland types	47	3.615	**0.035**
Below-ground species richness	Grazing	47	28.507	**<0.001**
	Grassland types	7	8.313	**0.014**
	Grazing: Grassland types	47	1.996	0.147

Table. S4. Summary of linear mixed effects models analyzing the effects of exclosure year on the grazing effects on EMF, multidiversity, above-ground diversity, and below-ground diversity (two-tailed statistical tests). Exclosure year was taken as fixed factor. Plots and sites nested within grassland types were taken as random factor. Log response ratios (LRRs) were used to examine the grazing effects on EMF, multidiversity, above-ground diversity, and below-ground diversity, respectively. Source data are provided as a Source Data file.

	Variable	DF	t-value	p-value
LRRs of EMF	Exclosure year	6	-1.364	0.221
LRRs of multidiversity	Exclosure year	6	-0.931	0.388
LRRs of above-ground diversity	Exclosure year	6	-1.029	0.343
LRRs of below-ground diversity	Exclosure year	6	-0.948	0.380

Table. S5. Summary of linear mixed models analyzing the relative importance of above- and below-ground diversity for EMF in ungrazed grasslands and grazed grasslands (two-tailed statistical tests). Above-ground diversity and below-ground diversity were taken as fixed factors. Plots and sites nested within grassland types were taken as random factor. The aboveground diversity and the numbers of functions beyond a given threshold (25%, 50%, and 75%) were standardized (min-max normalization) variables before the analysis. Source data are provided as a Source Data file.

EMF components	Variable	Ungrazed grasslands		Grazed grasslands	
		Parameter value	p-value	Parameter value	p-value
Average EMF	Above-ground diversity	0.203	**0.003**	0.004	0.932
	Below-ground diversity	-0.071	0.204	0.131	**0.022**
Weighted EMF	Above-ground diversity	0.171	**0.004**	0.012	0.758
	Below-ground diversity	-0.048	0.343	0.097	**0.036**
25% threshold EMF	Above-ground diversity	0.498	**0.009**	0.054	0.765
	Below-ground divesity	-0.158	0.334	0.206	0.352
50% threshold EMF	Above-ground diversity	0.790	**0.004**	-0.086	0.534
	Below-ground diversity	-0.098	0.682	0.441	**0.008**
75% threshold EMF	Above-ground diversity	0.268	0.228	-0.026	0.879
	Below-ground diversity	-0.211	0.364	0.163	0.519

Table. S6. Summary of linear mixed effects models analyzing the interactive effects of grazing and grassland types on ecosystem functions (two-tailed statistical tests). Grazing, grassland types, and their interaction were taken as fixed factors. Plot nested within sites were taken as random factor. AB = above-ground plant biomass; BB = below-ground root biomass; PN = plant community N; PP = plant community P; SAN = soil available N; SOC = soil organic C; MBC = microbial biomass C; MBN = microbial biomass N; Pathogen = Pathogen control; Mycorrhizal = Mycorrhizal mutualism. Source data are provided as a Source Data file.

	Variable	DF	F-value	p-value
AB	Grazing	47	156.693	**<0.0001**
	Grassland types	7	1.113	0.381
	Grazing: Grassland types	47	7.048	**0.002**
BB	Grazing	47	0.929	0.340
	Grassland types	7	0.846	0.469
	Grazing: Grassland types	47	1.762	**0.183**
PN	Grazing	47	24.687	**<0.0001**
	Grassland types	7	0.929	0.439
	Grazing: Grassland types	47	5.629	**0.006**
PP	Grazing	47	31.424	**<0.0001**
	Grassland types	7	1.448	0.298
	Grazing: Grassland types	47	6.338	**0.004**
SAN	Grazing	47	0.221	0.640
	Grassland types	7	2.136	0.189
	Grazing: Grassland types	47	2.747	0.074
SOC	Grazing	47	8.158	**0.006**
	Grassland types	7	2.496	0.152
	Grazing: Grassland types	47	2.472	**0.095**
MBC	Grazing	47	2.826	0.099
	Grassland types	7	3.013	0.114
	Grazing: Grassland types	47	5.972	**0.005**

MBN	Grazing	47	5.279	**0.026**
	Grassland types	7	1.039	0.403
	Grazing: Grassland types	47	0.483	0.620
Decomposers	Grazing	47	0.017	0.896
	Grassland types	7	0.287	0.759
	Grazing: Grassland types	47	0.636	0.534
Pathogen control	Grazing	47	3.145	0.083
	Grassland types	7	2.707	0.135
	Grazing: Grassland types	47	1.328	0.275
Mycorrhizal mutualism	Grazing	47	7.572	**0.008**
	Grassland types	7	0.897	0.450
	Grazing: Grassland types	47	2.329	0.109

Table. S7. Summary of linear mixed models analyzing the relative importance of above- and below-ground diversity for individual functions and possible combinations among functions in ungrazed grasslands and grazed grasslands (two-tailed statistical tests). Above-ground diversity and below-ground diversity were taken as fixed factors. Plots and sites nested within grassland types were taken as random factor. The aboveground diversity, individual functions, and the numbers of functions beyond a given threshold (25%, 50%, and 75%) were standardized (min-max normalization) variables before the analysis. AB = above-ground plant biomass; BB = below-ground root biomass; PN = plant community N; PP = plant community P; SAN = soil available N; SOC = soil organic C; MBC = microbial biomass C; MBN = microbial biomass N; Pathogen = Pathogen control; Mycorrhizal = Mycorrhizal mutualism. Source data are provided as a Source Data file.

Ecosystem functions	Variable	Ungrazed grasslands		Grazed grasslands	
		Parameter value	p-value	Parameter value	p-value
Average EMF	Above-ground diversity	0.203	**0.003**	0.004	0.932
	Below-ground diversity	-0.071	0.204	0.131	**0.022**
Above MF	Above-ground diversity	0.202	**0.029**	0.045	0.540
	Below-ground diversity	-0.069	0.376	0.218	**0.013**
Below MF	Above-ground diversity	0.199	**0.016**	0.003	0.958
	Below-ground divesity	-0.066	0.337	0.104	0.108
PMF	Above-ground diversity	0.226	0.142	-0.025	0.785
	Below-ground diversity	-0.190	0.163	0.218	0.076
NCMF	Above-ground diversity	0.225	**0.009**	-0.039	0.611
	Below-ground diversity	-0.161	**0.027**	0.178	**0.040**

AB	Above-ground diversity	0.197	0.287	0	1
	Below-ground diversity	0.020	0.902	0.005	0.943
BB	Above-ground diversity	0.282	0.260	0.218	0.202
	Below-ground diversity	-0.336	0.146	0.562	**0.019**
PN	Above-ground diversity	0.274	0.087	0.114	0.442
	Below-ground diversity	-0.212	0.140	0.505	**0.006**
PP	Above-ground diversity	0.141	0.114	0.047	0.606
	Below-ground diversity	-0.127	0.103	0.167	0.097
SAN	Above-ground diversity	0.134	0.225	-0.002	0.987
	Below-ground diversity	-0.042	0.653	0.169	0.245
SOC	Above-ground diversity	-0.046	0.772	-0.089	0.409
	Below-ground diversity	0.023	0.863	-0.035	0.766
MBC	Above-ground diversity	0.543	**0.015**	-0.141	0.247
	Below-ground diversity	-0.368	0.071	0.202	0.136
MBN	Above-ground diversity	0.467	**0.007**	0.031	0.825
	Below-ground diversity	-0.270	0.063	0.127	0.453

Decomposers	Above-ground diversity	0.362	**0.037**	0.217	0.151
	Below-ground diversity	-0.206	0.224	0.152	0.395
Pathogen control	Above-ground diversity	-0.114	0.556	0.004	0.971
	Below-ground diversity	0.136	0.492	-0.190	0.139
Mycorrhizal mutualism	Above-ground diversity	-0.117	0.505	-0.043	0.582
	Below-ground diversity	0.532	**0.001**	-0.059	0.529

Table. S8. Summary of linear mixed models analyzing the relative importance of bacterial diversity, fungal diversity, and protist diversity for individual functions and possible combinations among functions in ungrazed grasslands and grazed grasslands (two-tailed statistical tests). Bacterial diversity, fungal diversity, and protist diversity were taken as fixed factors. Plots and sites nested within grassland types were taken as random factor. The soil bacterial diversity, soil fungal diversity, soil protists diversity, and individual functions were standardized (min-max normalization) variables before the analysis. AB = above-ground plant biomass; BB = below-ground root biomass; PN = plant community N; PP = plant community P; SAN = soil available N; SOC = soil organic C; MBC = microbial biomass C; MBN = microbial biomass N; Pathogen = Pathogen control; Mycorrhizal = Mycorrhizal mutualism. Source data are provided as a Source Data file.

Ecosystem functions	Variable	Ungrazed grasslands		Grazed grasslands	
		Parameter value	p-value	Parameter value	p-value
Average EMF	Bacterial diversity	-0.054	0.291	0.026	0.595
	Fungal diversity	-0.026	0.451	-0.028	0.536
	Protist diversity	0.008	0.788	0.072	**0.004**
Above MF	Bacterial diversity	0.005	0.936	-0.007	0.926
	Fungal diversity	-0.075	0.118	0.124	0.093
	Protist diversity	0.030	0.480	0.075	**0.056**
Below MF	Bacterial diversity	-0.073	0.227	0.040	0.467
	Fungal diversity	-0.007	0.865	-0.085	0.098
	Protist diversity	0.004	0.921	0.072	**0.009**
PMF	Bacterial diversity	-0.068	0.552	0.103	0.346
	Fungal diversity	-0.119	0.151	0.050	0.617
	Protist diversity	-0.002	0.982	0.072	0.215

NCMF	Bacterial diversity	-0.031	0.621	0.061	0.439
	Fungal diversity	-0.026	0.518	-0.009	0.899
	Protist diversity	-0.058	0.122	0.086	**0.029**
AB	Bacterial diversity	0.144	0.267	0.039	0.562
	Fungal diversity	-0.110	0.235	0.002	0.975
	Protist diversity	0.122	0.150	-0.009	0.774
BB	Bacterial diversity	-0.225	0.255	0.058	0.782
	Fungal diversity	-0.090	0.538	0.278	0.164
	Protist diversity	-0.059	0.639	0.181	0.103
PN	Bacterial diversity	-0.020	0.865	-0.011	0.944
	Fungal diversity	-0.079	0.328	0.346	0.027
	Protist diversity	-0.036	0.622	0.158	**0.057**
PP	Bacterial diversity	-0.069	0.292	-0.071	0.436
	Fungal diversity	-0.051	0.238	0.071	0.398
	Protist diversity	-0.010	0.805	0.082	0.070
SAN	Bacterial diversity	0.013	0.872	0.019	0.886
	Fungal diversity	-0.007	0.889	-0.099	0.416
	Protist diversity	-0.011	0.817	0.122	0.065

	Bacterial diversity	0.037	0.735	-0.041	0.713
SOC	Fungal diversity	-0.021	0.772	0.170	0.102
	Protist diversity	0.017	0.793	-0.054	0.320
	Bacterial diversity	0.003	0.984	0.247	**0.039**
MBC	Fungal diversity	0	0.997	-0.192	0.080
	Protist diversity	-0.248	**0.020**	0.118	**0.042**
	Bacterial diversity	-0.190	0.145	0.159	0.281
MBN	Fungal diversity	-0.004	0.963	-0.231	0.096
	Protist diversity	-0.080	0.308	0.098	0.184
	Bacterial diversity	-0.252	0.133	-0.007	0.960
Decomposers	Fungal diversity	0.131	0.318	-0.362	**0.010**
	Protist diversity	-0.069	0.529	0.188	**0.011**
	Bacterial diversity	-0.010	0.949	0.077	0.500
Pathogen control	Fungal diversity	-0.251	**0.040**	-0.269	**0.014**
	Protist diversity	0.304	**0.005**	-0.031	0.573
	Bacterial diversity	-0.071	0.542	-0.142	0.086
Mycorrhizal mutualism	Fungal diversity	0.269	**0.001**	0.091	0.233
	Protist diversity	0.131	0.067	-0.015	0.713

Fig. S1.

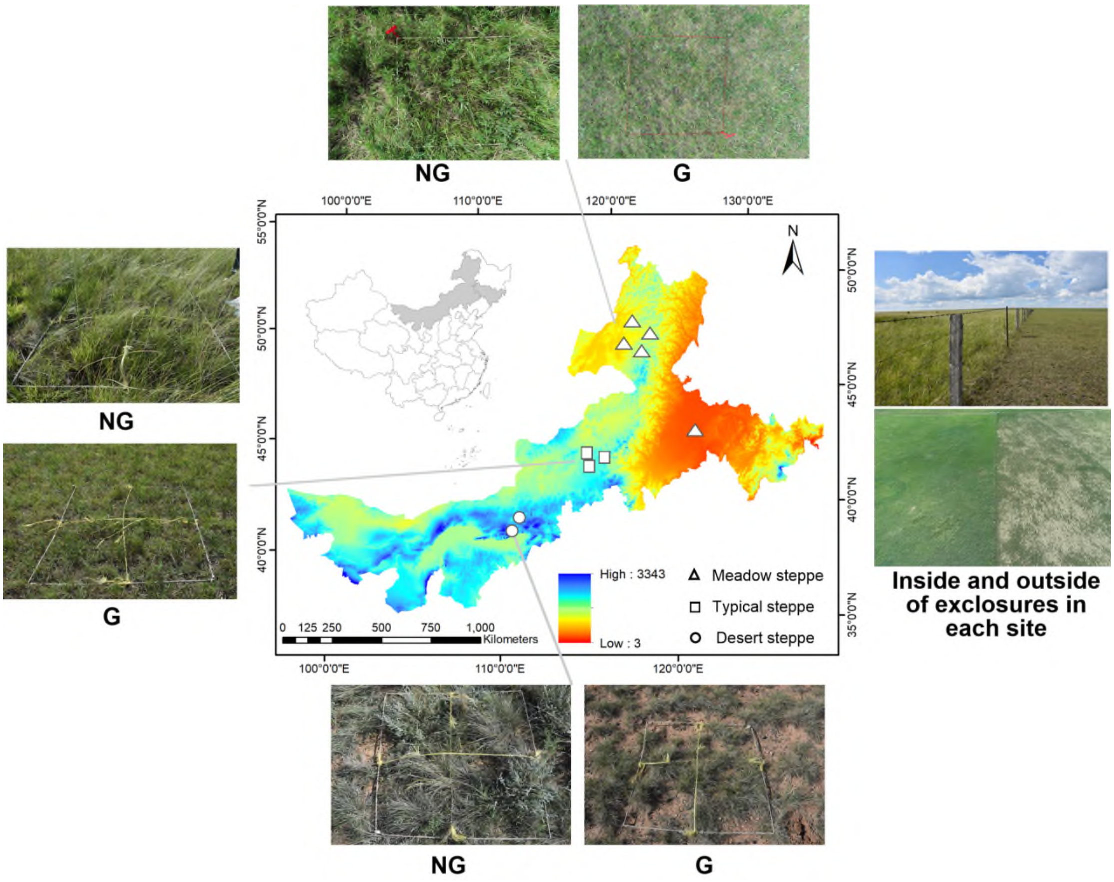

Figure S1. Distribution of sampling sites in northern China (sites are skewed to avoid overlap to clarify where different sites are located), and vegetation contrast inside and outside exclosures in the three grassland types: meadow steppe, typical steppe and desert steppe, NG, no grazing; G, grazing. Source data are provided as a Source Data file.

Fig. S2.

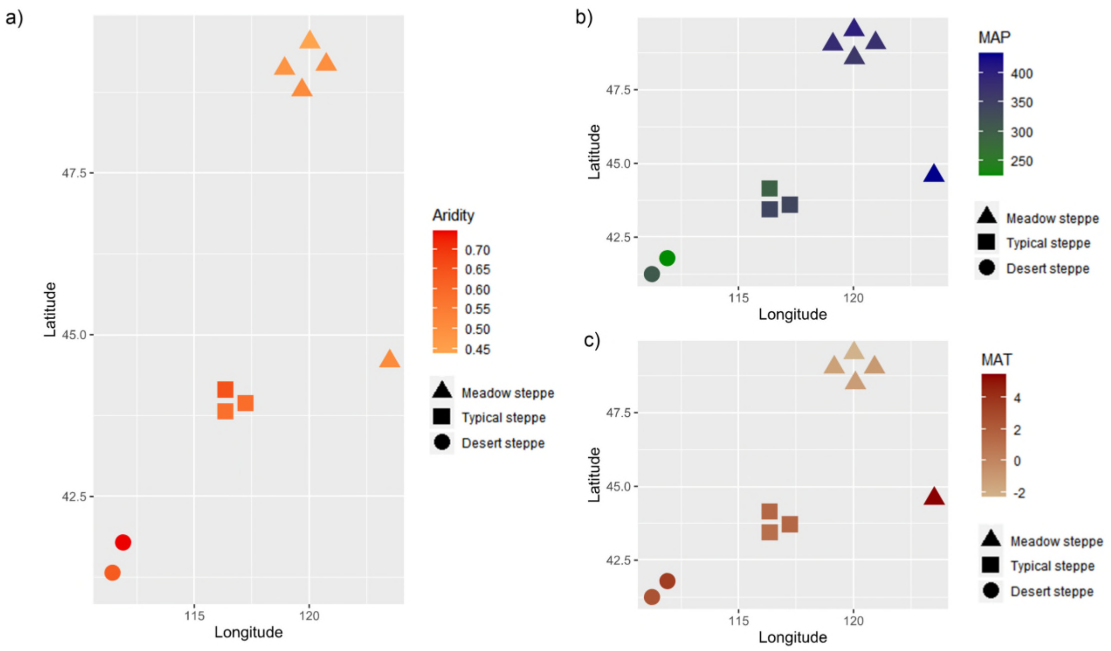

Figure S2. Aridity (a), precipitation (b), and temperature (c) across the sampling sites (sites are skewed to avoid overlap to clarify where different sites are located). MAP, mean annual precipitation; MAT, mean annual temperature. Source data are provided as a Source Data file.

Fig. S3.

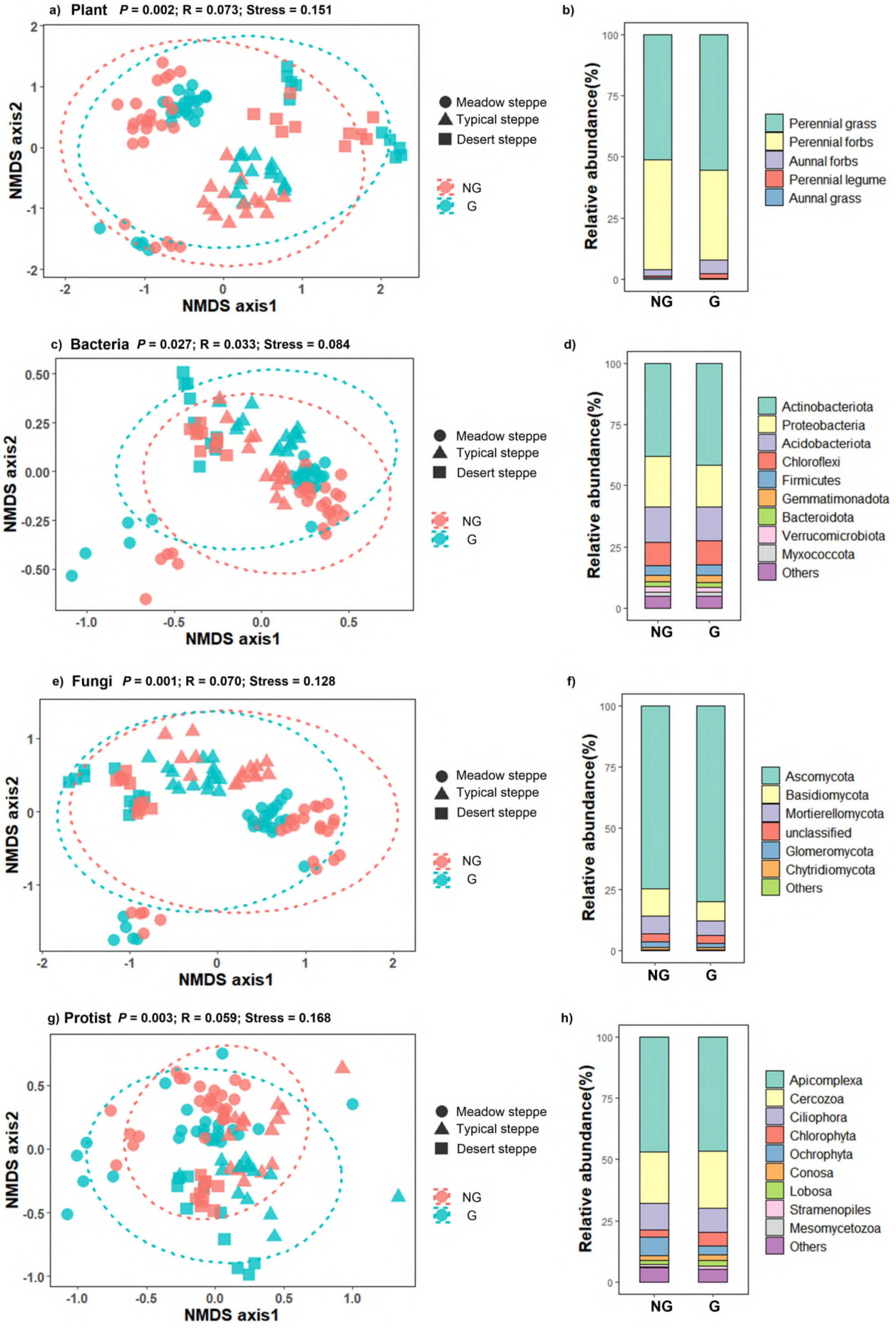

Figure S3. Non-metric multidimensional scaling (NMDS) analysis showed the community composition of (a) plant, (c) bacterial, (e) fungal, and (g) protist

communities in ungrazed and grazed grasslands. Similarity values were examined between ungrazed and grazed grasslands via the ANOSIM test, which are shown in each plot. And the relative abundances of phyla in (b) plant, (d) bacterial, (f) fungal, and (h) protist communities; NG, no grazing; G, grazing.

Fig. S4.

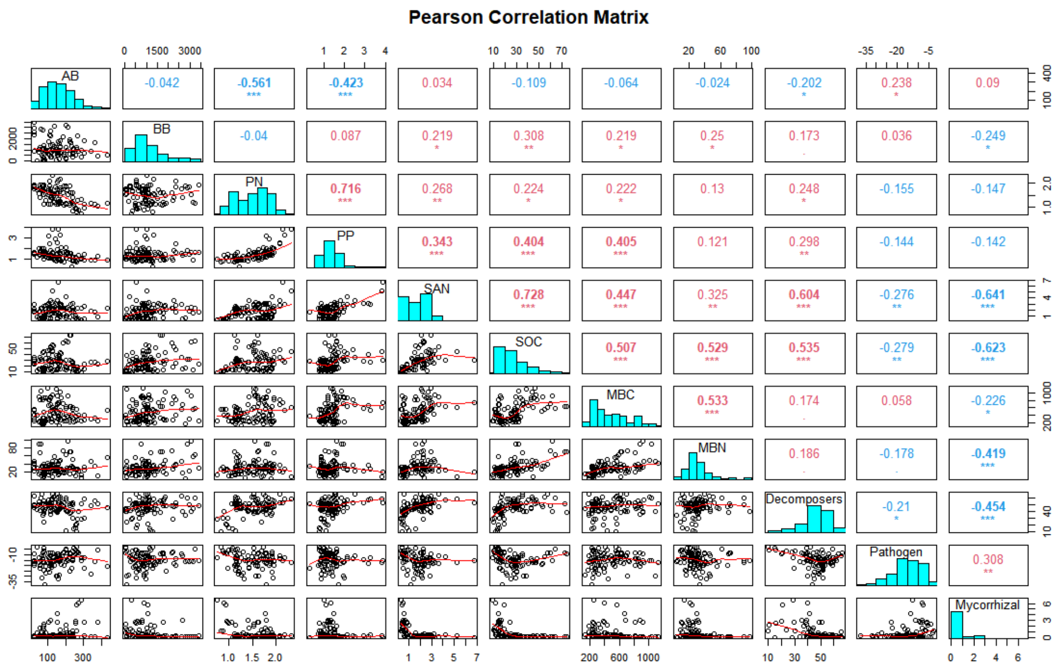

Figure S4. Scatterplots matrices for the 11 ecosystem functions. AB = above-ground plant biomass; BB = below-ground root biomass; PN = plant community N; PP = plant community P; SAN = soil available N; SOC = soil organic C; MBC = microbial biomass C; MBN = microbial biomass N; Pathogen = Pathogen control; Mycorrhizal = Mycorrhizal mutualism. The upper triangular matrix shows the pairwise relationships among functions (two-sided Pearson). P-values of the correlation coefficient are as follows: ***P<0.001, **P<0.01, *P<0.05. Source data are provided as a Source Data file.

Fig. S5.

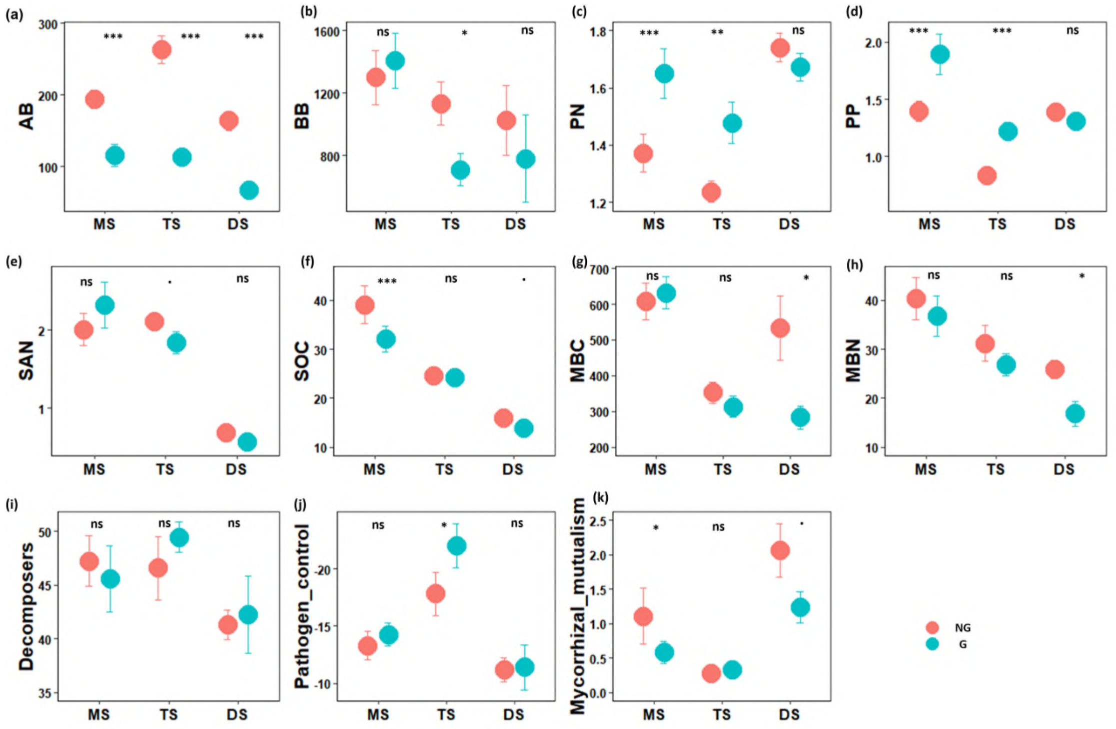

Figure S5. The effects of grazing on above-ground plant biomass (g/m^2) (a), below-ground root biomass (g/m^2) (b), plant community N (%) (c), plant community P (g/kg) (d); soil organic C (g/kg) (e), soil available N (mg/kg) (f); microbial biomass C (mg/kg), microbial biomass N (mg/kg) (h), decomposers (%) (i), Pathogen control (%) (j), mycorrhizal mutualism (%) (k). Dots with bars indicate means ± standard error (SE) (MS: n = 25; TS: n=15; DS: n=10). Statistical analysis was performed using linear mixed effects models with grazing, grassland types and their interaction as fixed factors, and plot nested within sites as random factors; The two-tailed statistical tests indicate significant effects by · $P < 0.1$; * $P < 0.05$; ** $P < 0.01$; *** $P < 0.001$. For exact statistical values, see Supplementary Table 6. NG, no grazing; G, grazing; MS, meadow steppe; TS, typical steppe; DS, desert steppe. Source data are provided as a Source Data file.

Fig. S6.

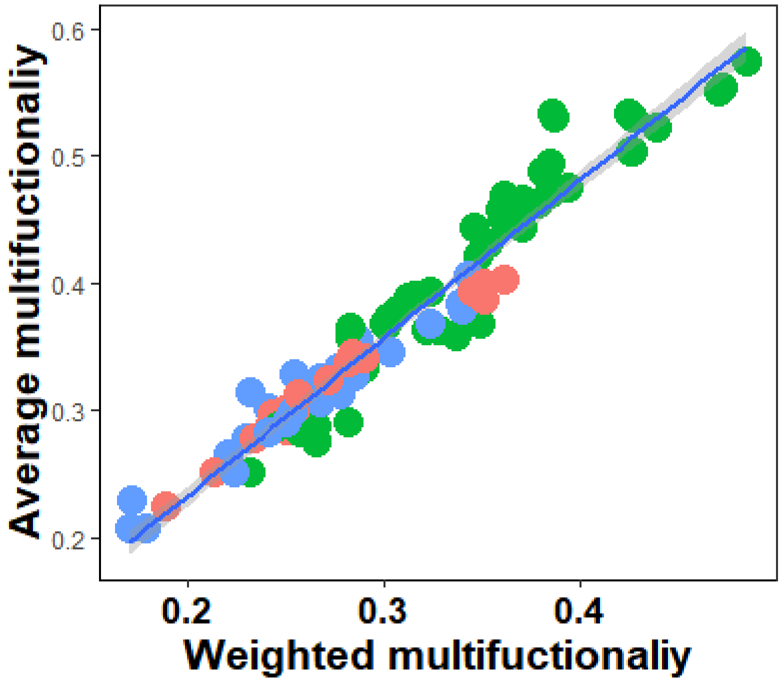

Figure S6. The relationships between average multifunctionality (EMF) and the weighted EMF (two-sided Pearson adjusted r-squared = 0.937, $p < 0.001$, n=100). The solid line represents the linear regression, while the gray shading indicates the 95% confidence interval. The weighted EMF is calculated as the average of all these functions after weighting of soil C, soil available N, plant communiy N, and plant communiy P with a weight of 0.5, so that these correlated variables have a combined weight of only 1; $P < 0.05$. Source data are provided as a Source Data file.

Fig. S7.

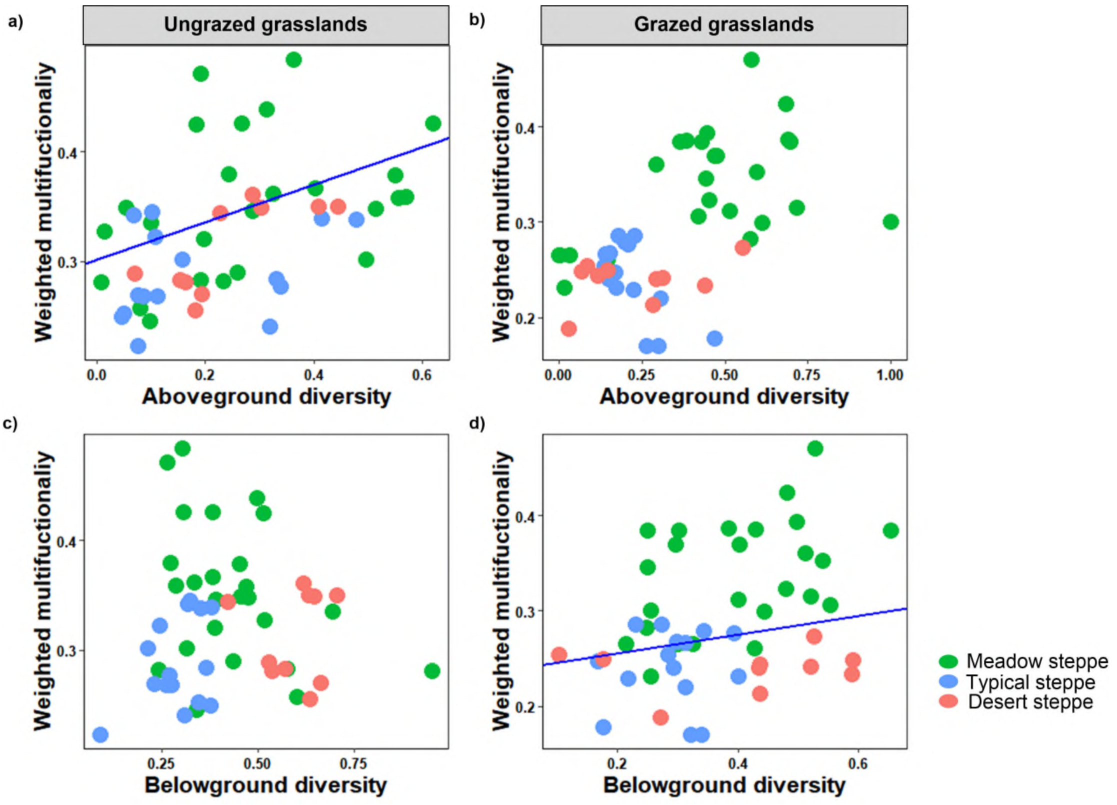

Figure S7. Relationships between aboveground, belowground diversity and weighted multifunctionaliy across ungrazeded (a, c) and grazed grasslands (b, d). The fitted lines are from the linear mixed effects model with above- and belowground diversity as fixed effects, and sites nested within grassland types as random effects; Statistical analysis was performed using linear mixed effects models with aboveground and belowground diversity as fixed factors, and plots and sites nested within grassland types as random factors ($P<0.05$; $n=50$); For exact statistical values, see Supplementary Table 5. The aboveground diversity was standardized (min-max normalization) variables before the analysis. Source data are provided as a Source Data file.

Fig. S8.

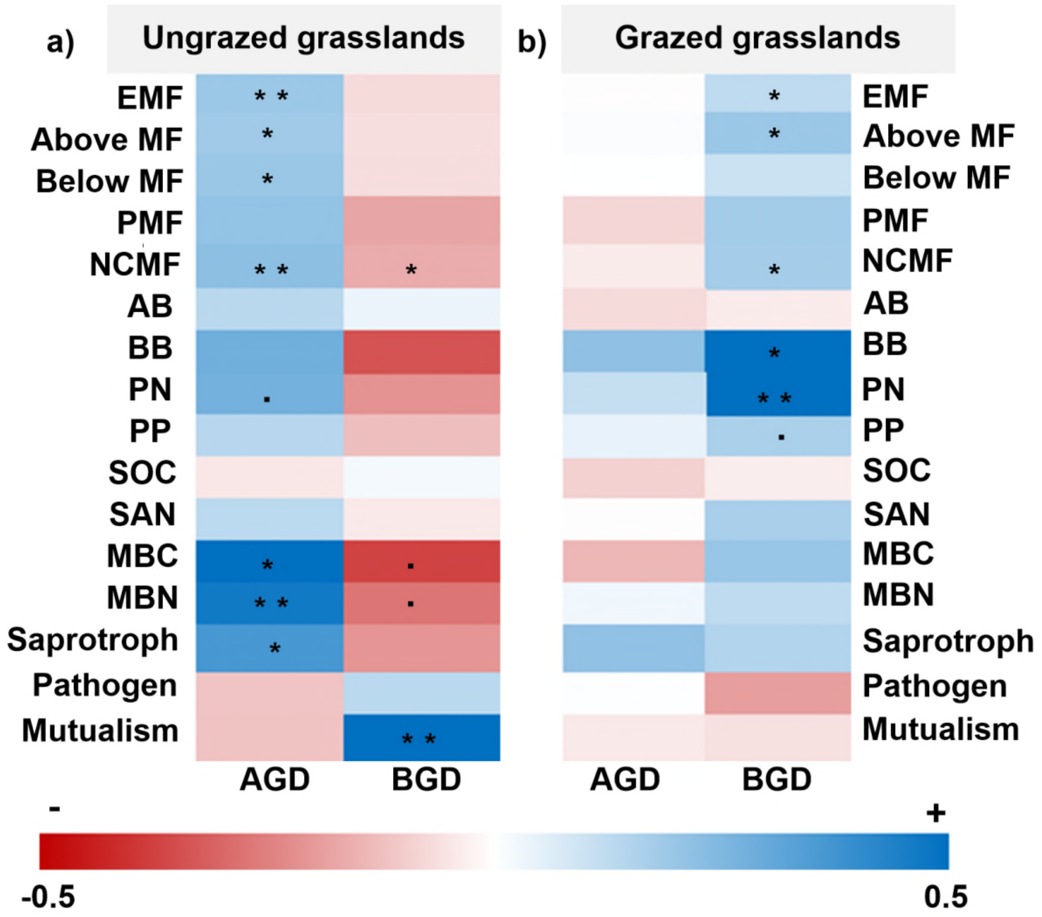

Figure S8 | Heatmap of correlation (linear mixed effects models) between above-, below-ground diversity and function combination. The numbers in the table are slope value. The shading from white to red represents gradation from low to high negative correlation. The shading from white to blue represents gradation from low to high positive correlation. EMF includes all the 11 functions. Above-ground MF (Above MF) includes plant above-ground biomass, and plant community N and P. Below-ground MF (Below MF) includes below-ground biomass, soil available N (SAN), soil organic C (SOC), Microbial biomass C (MBC), Microbial biomass C (MBN), Decomposers (saprotroph), Pathogen_control (pathogen), Mycorrhizal mutualism (mutualism). Production MF (PMF) includes above-ground plant biomass and below-ground plant biomass. Nutrient cycle MF (NCMF) includes soil available N (SAN), soil organic C (SOC), Microbial biomass C (MBC), microbial biomass C (MBN), and plant community N and P. AGD, aboveground diversity; BGD, belowground diversity. Statistical analysis was performed using linear mixed effects models with aboveground and belowground diversity as fixed factors, and plots and sites nested within grassland types as random factors (n=50); The two-tailed statistical tests indicate significant effects by · $P < 0.1$; * $P < 0.05$; ** $P < 0.01$. For exact statistical values, see Supplementary Table 7. The aboveground diversity and

individual functions were standardized (min-max normalization) variables before the analysis. Source data are provided as a Source Data file.

Fig. S9.

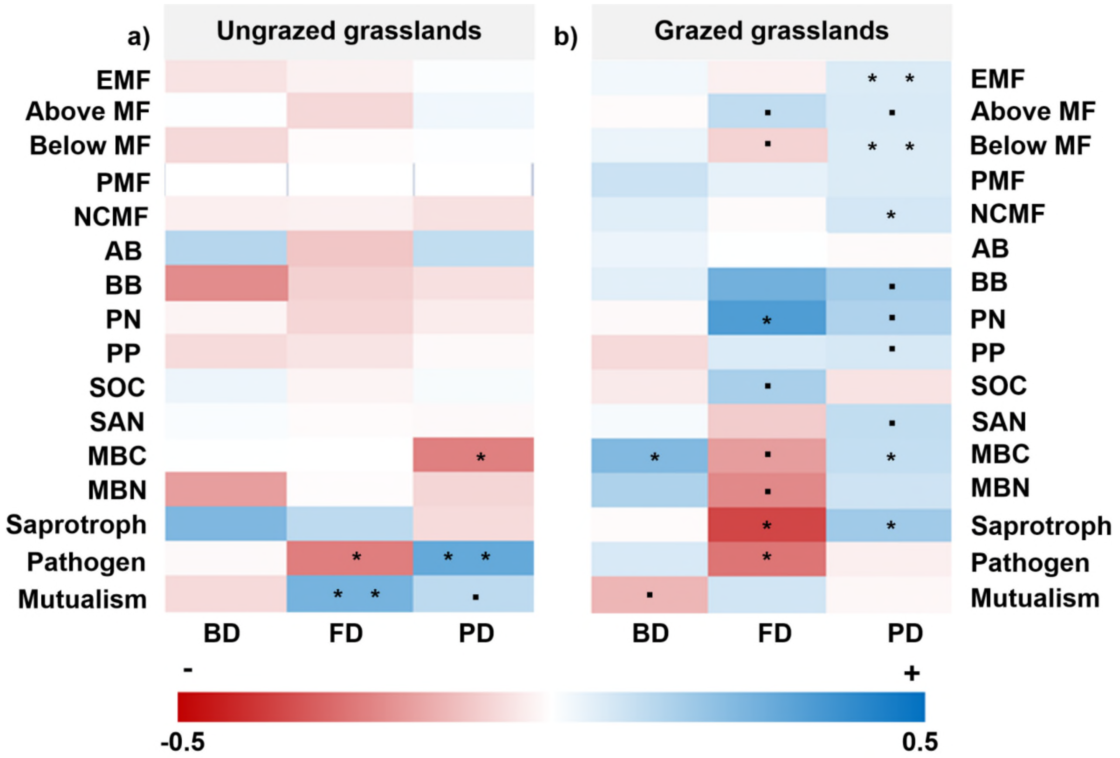

Figure S9 Heatmap of correlation (linear mixed effects models) between soil bacterial diversity, fungal diversity, protist diversity and function combination. The numbers in the table are slope value. The shading from white to blue represents gradation from low to high positive correlation. The shading from white to red represents gradation from low to high negative correlation. EMF includes all the 11 functions. Above-ground MF (Above MF) includes plant above-ground biomass, and plant community N and P. Below-ground MF (Below MF) includes below-ground biomass, soil available N (SAN), soil organic C (SOC), Microbial biomass C (MBC), Microbial biomass C (MBN), Decomposers (saprotroph), Pathogen_control (pathogen), Mycorrhizal mutualism (mutualism). Production MF (PMF) includes above-ground plant biomass and below-ground plant biomass. Nutrient cycle MF (NCMF) includes soil available N (SAN), soil organic C (SOC), Microbial biomass C (MBC), microbial biomass C (MBN), and plant community N and P. BD-bacterial diversity; FD-fungal diversity; PD-protist diversity. Statistical analysis was performed using linear mixed effects models with bacterial diversity, fungal diversity, and protist diversity as fixed factors, and plots and sites nested within grassland types as random

factors (n=50); The two-tailed statistical tests indicate significant effects by · $P < 0.1$; * $P < 0.05$; ** $P < 0.01$. For exact statistical values, see Supplementary Table 8. The soil bacterial diversity, soil fungal diversity, soil protists diversity, and the individual functions were standardized (min-max normalization) variables before the analysis. Source data are provided as a Source Data file.

Fig. S10.

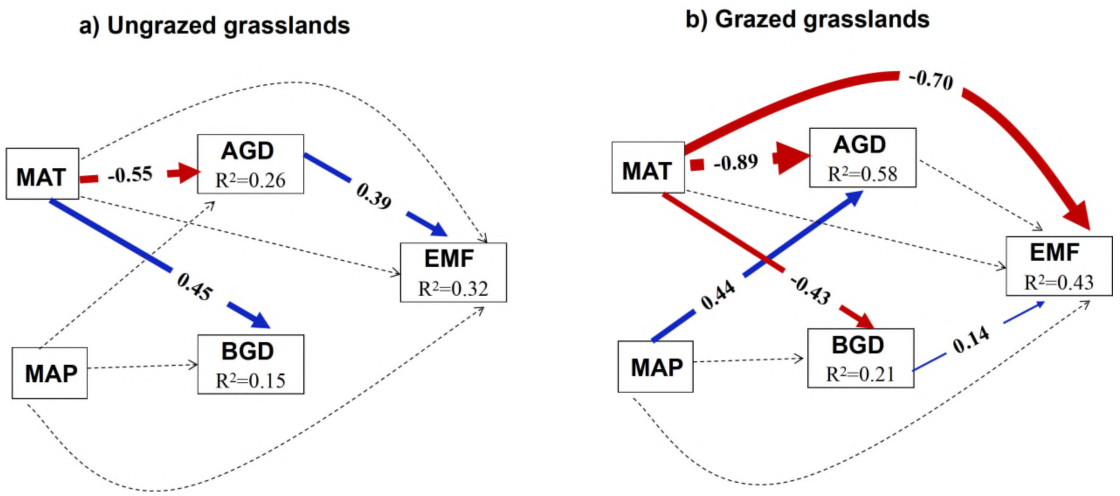

Figure S10. Structural equation models with mean annual temperature, mean annual precipitation, above-ground diversity, and below-ground diversity as predictors of EMF for ungrazed (a) and grazed (b) grasslands. Blue and red solid arrows indicate positive and negative effects, respectively, and grey arrows indicate non-significant paths ($P > 0.1$). The thickness of the arrows reflects the magnitude of the standardized SEM coefficients. There was non-significant deviation of the data from the models (Ungrazed: P=0.245; Grazed: P = 0.614). MAT, mean annual temperature; MAP, mean annual precipitation; AGD, aboveground diversity; BGD, belowground diversity. The aboveground diversity was standardized (min-max normalization) variables before the analysis. Source data are provided as a Source Data file.

Fig. S11.

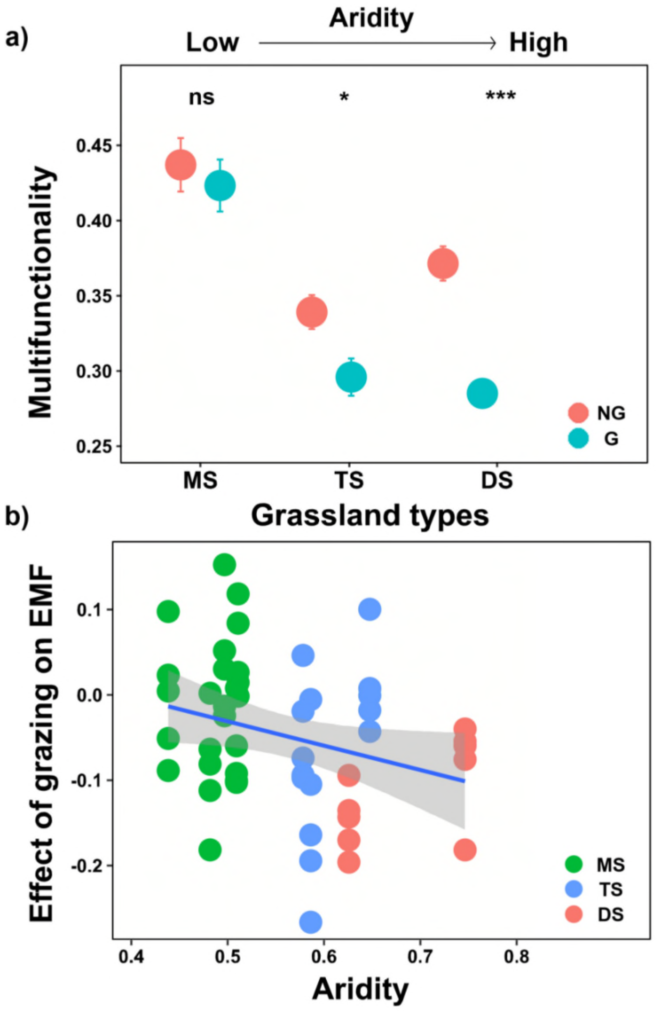

Figure S11 | The long-term effects of livestock grazing on multifunctionality across aridity gradient including three types of grasslands. a) Difference in multifunctionality inside (ungrazed) and outside (grazed) exclosure in three types of grasslands. Dots with bars indicate means ± standard error (SE) (MS: n = 25; TS: n=15; DS: n=10). Statistical analysis was performed using linear mixed effects models with grazing, grassland types and their interaction as fixed factors, and plot nested within sites as random factors (Grazing: DF=47, F=16.277, P<0.001; Grassland types: DF=7, F=3.94, P=0.071; Grazing:Grassland types: DF=47, F=4.545, P=0.016); The two-tailed statistical tests indicate significant effects by *P < 0.05; ***P < 0.001; ns, nonsignificant. NG, no grazing; G, grazing. b) Relationships between aridity and the effects of grazing on multifunctionality (two-sided Pearson adjusted r-squared = 0.062, p = 0.045). The solid line represents the linear regression, while the gray shading indicates the 95% confidence interval. Log response ratios (LRRs) compare multifunctionaliy outside and inside exclosures were used to quantify the effects of grazing on multifunctionality. MS, meadow steppe; TS, typical

steppe; DS, desert steppe; EMF, ecosystem multifunctionality. Considering the annual variations of biomass production can be large due to fluctuating environmental factors such as precipitation and temperature, EMF was calculated with the average above-ground biomass of 2018 and 2020. Source data are provided as a Source Data file.

Fig. S12.

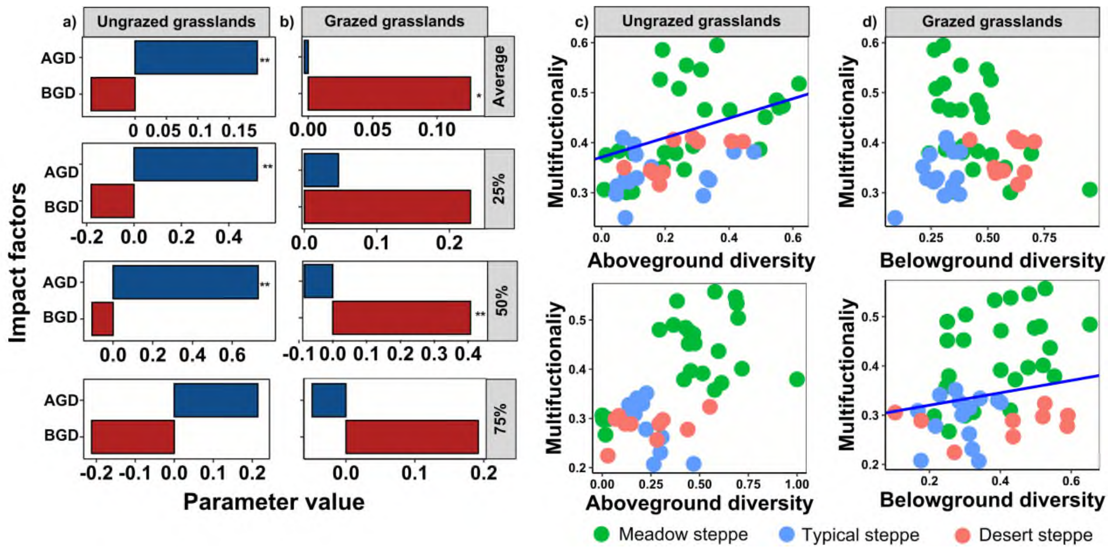

Figure S12 | The relative strength of above- and below-ground diversity in supporting multifunctionality in ungrazed grasslands and long-term grazed grasslands. The relative strength of above- and below-ground diversity for average multifunctionality and multithreshold functioning (the number of functions above multiple thresholds) in ungrazed grasslands (a) and grazed grasslands (b). And the fitted linear relationships between average multifunctionality and above-ground diversity and below-ground diversity in ungrazed grasslands (c) and grazed grasslands (d). All statistical analysis was performed using linear mixed effects models with aboveground and belowground diversity as fixed factors, and plots and sites nested within grassland types as random factors (Ungrazed grasslands: Average, the p value of AGD is P = 0. 004, the p value of BGD is P = 0. 218; 25%, the p value of AGD is P = 0. 005, the p value of BGD is P = 0. 275; 50%, the p value of AGD is P = 0. 004, the p value of BGD is P = 0. 612; 75%, the p value of AGD is P = 0. 242, the p value of BGD is P = 0. 354; Grazed grasslands: Average, the p value of AGD is P = 0. 947, the p value of BGD is P = 0. 028; 25%, the p value of AGD is P = 0. 792, the p value of BGD is P = 0. 296; 50%, the p value of AGD is P = 0. 484, the p value of BGD is P = 0. 006; 75%, the p value of AGD is P = 0. 774, the p value of BGD is P = 0. 438; n=50); The two-tailed statistical tests indicate significant effects by *P < 0.05; **P < 0.01. AGD, above-ground diversity; BGD, below-ground diversity; Average, the average EMF; 25%, 50%, and 75%, the number of functions beyond 25%, 50%, and 75% threshold. The aboveground diversity and the numbers of functions beyond a given threshold (25%, 50%, and 75%) were standardized (min-max normalization) variables before the analysis. Considering the annual variations of biomass production can be large due to fluctuating environmental factors such as precipitation and temperature, multifunctionaliy was calculated with the average above-ground biomass

of 2018 and 2020. Source data are provided as a Source Data file.
