# Supplementary material for: Experimental impacts of grazing on grassland biodiversity and function are explained by aridity
Source: Nat Commun. 2023 Aug 19;14:5040. doi: 10.1038/s41467-023-40809-6 (PMC10439935; doi:10.1038/s41467-023-40809-6)
Supplement: Supplementary file 2 — Reporting Summary [file 41467_2023_40809_MOESM2_ESM.pdf]

nature portfolio

Corresponding author(s): Ling Wang

Last updated by author(s): Jul 20, 2023

Reporting Summary

Nature Portfolio wishes to improve the reproducibility of the work that we publish. This form provides structure for consistency and transparency in reporting. For further information on Nature Portfolio policies, see our Editorial Policies and the Editorial Policy Checklist.

Statistics

For all statistical analyses, confirm that the following items are present in the figure legend, table legend, main text, or Methods section.

n/a	Confirmed	
☐	☒	The exact sample size (*n*) for each experimental group/condition, given as a discrete number and unit of measurement
☐	☒	A statement on whether measurements were taken from distinct samples or whether the same sample was measured repeatedly
☐	☒	The statistical test(s) used AND whether they are one- or two-sided<br>*Only common tests should be described solely by name; describe more complex techniques in the Methods section.*
☒	☐	A description of all covariates tested
☐	☒	A description of any assumptions or corrections, such as tests of normality and adjustment for multiple comparisons
☐	☒	A full description of the statistical parameters including central tendency (e.g. means) or other basic estimates (e.g. regression coefficient) AND variation (e.g. standard deviation) or associated estimates of uncertainty (e.g. confidence intervals)
☐	☒	For null hypothesis testing, the test statistic (e.g. *F*, *t*, *r*) with confidence intervals, effect sizes, degrees of freedom and *P* value noted<br>*Give P values as exact values whenever suitable.*
☒	☐	For Bayesian analysis, information on the choice of priors and Markov chain Monte Carlo settings
☒	☐	For hierarchical and complex designs, identification of the appropriate level for tests and full reporting of outcomes
☐	☒	Estimates of effect sizes (e.g. Cohen's *d*, Pearson's *r*), indicating how they were calculated

Our web collection on statistics for biologists contains articles on many of the points above.

Software and code

Policy information about availability of computer code

Data collection	Climatic variable were collected from the WorldClim global database (https://www.worldclim.org/) using R software (version 4.1.0), and the code have been deposited in the Figshare database (https://figshare.com/s/a368cc6f2de4f1e0d39d).
Data analysis	All analysis were performed using R software (version 4.1.0), including the package of nlme , piecewiseSEM, and pairwiseAdonis. R scripts used for data analysis have been deposited in the Figshare database (https://figshare.com/s/a368cc6f2de4f1e0d39d).

For manuscripts utilizing custom algorithms or software that are central to the research but not yet described in published literature, software must be made available to editors and reviewers. We strongly encourage code deposition in a community repository (e.g. GitHub). See the Nature Portfolio guidelines for submitting code & software for further information.

Data

Policy information about availability of data

All manuscripts must include a data availability statement. This statement should provide the following information, where applicable:

- Accession codes, unique identifiers, or web links for publicly available datasets
- A description of any restrictions on data availability
- For clinical datasets or third party data, please ensure that the statement adheres to our policy

All Bacterial, fungal and protist sequences have been deposited in NCBI's SRA database under project accession numbers PRJNA995873. All data that support the findings of this study are available in the Figshare database (https://figshare.com/s/a368cc6f2de4f1e0d39d). The mean annual temperature, mean annual precipitation, and aridity level of each site using datafrom the WorldClim global database (https://www.worldclim.org/). Source data are provided with this paper.

Human research participants

Policy information about studies involving human research participants and Sex and Gender in Research.

Reporting on sex and gender	N/A
Population characteristics	N/A
Recruitment	N/A
Ethics oversight	N/A

Note that full information on the approval of the study protocol must also be provided in the manuscript.

Field-specific reporting

Please select the one below that is the best fit for your research. If you are not sure, read the appropriate sections before making your selection.

☐ Life sciences ☐ Behavioural & social sciences ☒ Ecological, evolutionary & environmental sciences

For a reference copy of the document with all sections, see nature.com/documents/nr-reporting-summary-flat.pdf

Ecological, evolutionary & environmental sciences study design

All studies must disclose on these points even when the disclosure is negative.

Study description	This study is based on a standardized field survey includes paired grazing plots (including and excluding livestock) of 10 locations from 3 types of grasslands, where we collected composite topsoil samples and plant samples. The selected sites experienced decades of grazing, and the exclusion of livestock was done over 10 years at each site. Based on this survey, we aim to compare levels of biodiversity and multifunctionality in grazed and ungrazed grasslands, and examine the relationship between biodiversity and multifunctionality.
Research sample	Samples have been collected from ungrazed and grazed grasslands across 10 experimental sites including three different types of grasslands situated along a 1100 km transect from east to west including meadow steppes, typical steppes and desert steppes across an aridity gradient (from less to more arid). These sites represent three major grassland types including the most dominant vegetation characteristics found in northern China. The samples were selected to cover the entire biogeographic range along with a broad range of environmental gradients.
Sampling strategy	We choose 10 geographically distinct sites to get the aridity gradients within this region. At each site, a pair of sampling area (50 m × 50 m) was selected randomly on both sides of the fence, and 5 1 m × 1 m plots (5 replicates for control including grazing and 5 replicates for grazing exclusion) were set at the four corners and the center of the area, which has been estimated to be a good compromise between enough space to encompass the variability in plant and soil community based on the literature. Our samples were selected to cover the entire biogeographic range along with a broad range of environmental gradients in northern China (Supplementary Fig. 1 and Table 1)
Data collection	Data collection on site was mainly recorded by GL and MZ, with the help of YW and YX. Above-ground biomass was clipped at the ground level and oven dried at 65°C for 48 h. Then it was weighed and ground into a fine powder on a ball mill for plant community nitrogen and phosphorus analyses. Soil samples were collected by taking five soil cores (2.5-cm diameter) at 10 cm depth in each of the five 1 × 1 m plots at each site. The five soil cores were mixed in situ to form one composite sample representing each plot. After removing the rocks and roots, the soil was passed through a 2-mm-mesh sieve and separated into two parts. One part was air-dried and used to determine soil organic C. The other part was kept in a freezer (MOBICOOL CoolFreeze CF-50) to maintain a temperature of -18°C and carried back to the laboratory as soon as possible for soil microbial community analysis and microbial biomass C, N and available nitrogen analysis. We then collected belowground root biomass to a depth of 30 cm using soil cores (diameter 7 cm) in each of these five quadrats as well. Roots were collected by rinsing the samples using sieves (mesh size 0.25 mm) at the same day, and

	then oven dried at 65°C for 48 h and weighed. MZ, GL and YW completed analyses of samples in the laboratory and collected data. Soil organic C was determined with the K2Cr2O7 titration method after digestion (Nelson and Sommers 1975). Soil microbial biomass carbon and microbial biomass nitrogen were measured by chloroform fumigation-extraction method (Vance et al., 1987). Soil NH4+ and NO3- were analyzed using an Alliance Flow Analyzer (Alliance Flow Analyzer, Futura, frépillon, France). Soil available N was determined as the sum of ammonium and nitrate. Plant community N content was measured using the CHNOS Elemental Analyzer (vario EL cube), and phosphorus content was analysed using fully automated high technology discrete analyzer (Smartchem 450, AMS, France) after H2SO4-H2O2 digestion.
Timing and spatial scale	Samples have been collected from ungrazed grazed grasslands across 10 experimental sites including three different types of grasslands situated along a 1100 km transect from east to west including meadow steppes, typical steppes and desert steppes across an aridity gradient (from less to more arid). Plant and soil sampling were carried out during the summer (late July to August) of 2020, corresponding to annual peak-standing biomass.
Data exclusions	No data were excluded from the analyses.
Reproducibility	All codes needed to reproduce our results are available, and all data is open source.
Randomization	This is not relevant to our study as it is a field experiment.
Blinding	To insure blinding, all the samples were analyzed randomly.

Did the study involve field work? ☒ Yes ☐ No

Field work, collection and transport

Field conditions	This study was conducted in dry grasslands from Northern China (111.23 E to 123.51 E, 41.25 N to 49.52 N) ranging from arid to semi-arid drylands. Mean annual precipitation varied from 225 mm to 402 mm, mean annual air temperature ranged from -2.3°C to 6.1°C, and the aridity ranged from 0.438 to 0.746.
Location	1.Latitude：49.52；Longitude：120.03；Aridity：0.438<br>2.Latitude：49.35；Longitude：120.13；Aridity：0.482<br>3.Latitude：49.33；Longitude：120.05；Aridity：0.497<br>4.Latitude：44.59；Longitude：123.51；Aridity：0.509<br>5.Latitude：48.50；Longitude：119.68；Aridity：0.511<br>6.Latitude：43.55；Longitude：116.69；Aridity：0.578<br>7.Latitude：43.60；Longitude：116.75；Aridity：0.586<br>8.Latitude：44.15；Longitude：116.35；Aridity：0.648<br>9.Latitude：41.25；Longitude：111.23；Aridity：0.626<br>10. Latitude：41.79；Longitude：111.90；Aridity：0.746
Access & import/export	All plant and soil samples were collected by our co-authors under permits of local governments.
Disturbance	This study did not cause any environmental disturbnace.

Reporting for specific materials, systems and methods

We require information from authors about some types of materials, experimental systems and methods used in many studies. Here, indicate whether each material, system or method listed is relevant to your study. If you are not sure if a list item applies to your research, read the appropriate section before selecting a response.

Materials & experimental systems

n/a	Involved in the study
☒	☐ Antibodies
☒	☐ Eukaryotic cell lines
☒	☐ Palaeontology and archaeology
☒	☐ Animals and other organisms
☒	☐ Clinical data
☒	☐ Dual use research of concern

Methods

n/a	Involved in the study
☒	☐ ChIP-seq
☒	☐ Flow cytometry
☒	☐ MRI-based neuroimaging
